# *Erwinia amylovora* Expresses Fast and Simultaneously *hrp*/*dsp* Virulence Genes during Flower Infection on Apple Trees

**DOI:** 10.1371/journal.pone.0032583

**Published:** 2012-03-06

**Authors:** Doris Pester, Renáta Milčevičová, Johann Schaffer, Eva Wilhelm, Sylvia Blümel

**Affiliations:** 1 Institute of Plant Health, Austrian Agency for Health and Food Safety (AGES), Vienna, Austria; 2 Health and Environment, Austrian Institute of Technology (AIT), Seibersdorf, Austria; University of Wisconsin-Milwaukee, United States of America

## Abstract

**Background:**

Pathogen entry through host blossoms is the predominant infection pathway of the Gram-negative bacterium *Erwinia amylovora* leading to manifestation of the disease fire blight. Like in other economically important plant pathogens, *E. amylovora* pathogenicity depends on a type III secretion system encoded by *hrp* genes. However, timing and transcriptional order of *hrp* gene expression during flower infections are unknown.

**Methodology/Principal Findings:**

Using quantitative real-time PCR analyses, we addressed the questions of how fast, strong and uniform key *hrp* virulence genes and the effector *dspA/E* are expressed when bacteria enter flowers provided with the full defense mechanism of the apple plant. In non-invasive bacterial inoculations of apple flowers still attached to the tree, *E. amylovora* activated expression of key type III secretion genes in a narrow time window, mounting in a single expression peak of all investigated *hrp/dspA/E* genes around 24–48 h post inoculation (hpi). This single expression peak coincided with a single depression in the plant *PR-1* expression at 24 hpi indicating transient manipulation of the salicylic acid pathway as one target of *E. amylovora* type III effectors. Expression of *hrp/dspA/E* genes was highly correlated to expression of the regulator *hrpL* and relative transcript abundances followed the ratio: *hrpA*>*hrpN*>*hrpL*>*dspA/E*. Acidic conditions (pH 4) in flower infections led to reduced virulence/effector gene expression without the typical expression peak observed under natural conditions (pH 7).

**Conclusion/Significance:**

The simultaneous expression of *hrpL*, *hrpA*, *hrpN*, and the effector *dspA/E* during early floral infection indicates that speed and immediate effector transmission is important for successful plant invasion. When this delicate balance is disturbed, e.g., by acidic pH during infection, virulence gene expression is reduced, thus partly explaining the efficacy of acidification in fire blight control on a molecular level.

## Introduction

The plant disease fire blight is caused by the Gram-negative bacterium *Erwinia amylovora* and is of recurring concern in pome fruit production. Economically relevant host plants include apple, pear and quince but many ornamentals of the Rosaceae family become infected as well [1]. Either blossoms, shoots or the rootstock can show blight symptoms leading to severe economic losses at varying extent. Large scale spreading of fire blight is ascribed to unintended trade with latent infected plants, whereas regional dissemination is due to pollinating insects, rain and wind [2]. Notably, new manifestations of the fire blight disease occur predominantly after blossoms of host plants were infected [2]. Thus, blossom infection plays an important role in gain of new geographical areas infested by the pathogen.

On host flowers, *E. amylovora* first multiplies on the stigmatic surface [3]. At high humidity, the bacteria enter the flower tissue through the nectarthodes located in the floral cup [4]. Although invasion of flowers is responsible for primary contamination with fire blight and substantially contributes to epidemics [2], molecular knowledge about the early infection process regarding bacterial virulence gene expression is absent. Regarding responsive plant gene expression, knowledge is limited to studies from leaves, stems, shoots, *in vitro* plantlets or immature fruits [5], [6], [7], [8], [9], [10], [11]. Only one study investigated the plant gene expression in detached flowers upon *E. amylovora* inoculation, but not bacterial gene expression [12]. The type III secretion system is an essential pathogenicity determinant during the early infection process in many phytopathogenic bacteria [13]. Plant pathogens such as *Pseudomonas syringae* and *Xanthomonas campestris* exploit natural openings on leaves, e.g., stomata or hydathodes for infection and manipulate the plant defense system with type III secreted effector proteins [14]. Also in *E. amylovora* the type III secretion system was shown to be essential for floral as well as shoot infections [8], [15]. Main efforts to understand the early infection process by *E. amylovora* are based on studies in vegetative plant parts or immature pear, where infection has to be artificially assisted by wounding the plant [6], [9], [10], [11]. However, the time point when *E. amylovora* genetically activates its type III secretion system, especially if not assisted by wounding and in the presence of the full plant defense such as in infections of flowers attached to the tree, still remains to be elucidated.

The type III secretion system consists of structural, regulatory and effector components and allows pathogenic bacteria to transmit effectors into the host cell [13]. In *E. amylovora*, the *hrp* genes (for hypersensitive reaction and pathogenicity) encode the components of the type III secretion system [15] and their expression is directly linked to virulence. The master switch for expression of this system is HrpL, an alternative sigma 70 factor, which can bind to the *hrp*-Box promoter elements present in all *hrp-* and *dsp*-genes [16], [17]. The protein channel for effector transmission is composed of the pilin HrpA [18], [19] and supports secretion of two functionally well characterized proteins, the harpin HrpN and the effector DspA/E. HrpN was initially isolated as elicitor of the hypersensitive response reaction (HR) in non-host tobacco [20], [21]. In host plants, HrpN was shown to be secreted along the pilus into the apoplast [18], [22], where it probably forms pores in the plant plasma membrane and functions as the main translocator protein [23], [24]. In support of this, HrpN proved to be necessary for efficient translocation of the effector DspA/E into plant cells [25]. DspA/E (for disease-specific) is absolutely required for *E. amylovora* virulence with mutants being apathogenic [26], [27], [28], [29]. In the plant cell, DspA/E putatively interacts with specific host plant receptor-like serine/threonine kinases, thereby interfering with plant signaling [30]. These findings are in line with the inability of *dspA/E* mutants to effectively suppress salicylic-acid (SA)-activated plant immunity, such as callose deposition [31], [32]. On the other hand, previous studies investigating the host transcriptional response upon *E. amylovora* inoculation did not find evidence for a differential expression of the pathogenesis-related protein 1 (*PR*-*1*), which would indicate an influence on the SA-mediated plant response [31], [33], [34]. This is astonishing, since the SA-mediated plant immunity is one of the major targets manipulated by type III effectors either directly or indirectly [31], [35], [36]. The *E. amylovora* DspA/E influences SA-dependent callose deposition [31], [32] and thus would be a good candidate effector involved in manipulation of the plant SA-signaling. In this context, expression of *dspA/E* itself showed a transient peak in *E. amylovora* populations growing epiphytically on flowers [37] and a similar transcriptional induction upon inoculation on immature pear fruit [11]. Thus, one might expect a transient effect on the plant defense system as well. However, the timing of *dspA/E* expression relative to *hrp* gene expression during the development of infection remains to be determined [11], [38].

The de-novo assembly of the type III secretion is energy consuming. This is why bacteria tightly restrict its expression until conditions arise which suggest host proximity [39]. In *E. amylovora*, these conditions include low nutrients, low temperature and low pH and generally resemble the plants apoplast environment [10], [29]. The inducing effect of acidic pH 5.5 on *hrp* gene expression [10] is particularly interesting regarding the use of acidifying products in fire blight control to prevent flower infections. Acidic stone meal or antagonistic yeast formulations with a pH around 4.0 were shown to inhibit pathogen growth [40]. On the other hand, *E. amylovora* strains which tolerate more acidity for growth are also described as more virulent (research report of project no 100448; www.dafne.at). It is currently not known if the inducing effect of acidic pH on *hrp* gene expression is balanced by the negative effect on pathogen growth at pH 4.0 and how this affects virulence.

We report here the temporal expression pattern of key genes for *E. amylovora* type III secretion for the first time during non-invasive bacterial inoculations on apple flowers still attached to the tree. The quantity and timing of *hrp* gene expression was determined by newly established quantitative real-time PCR analyses and compared to the expression of a virulence factor not involved in type III secretion, the amylovoran synthesis gene *amsG*. Parallel to *hrp* gene expression, expression of two host defense genes, *PR-1* and *MalMir1*, was monitored in the same flower tissues to assess plant defense response. Since acidification is relevant for fire blight control, the influence of acidic pH 4.0 on *hrp* expression was tested as well and compared to neutral pH.

## Materials and Methods

### Apple flower-*E. amylovora* inoculations

Freshly opened flowers of two year-old potted *Malus domestica* ‘Golden Delicious’ were manually inoculated with *E. amylovora* 295/93 (deposited as CFBP 6449 in the French culture collection) by a non-invasive technique. For inoculation, liquid overnight cultures were resuspended in water buffered either with piperazine-1,4-bis(2-ethanesulfonic acid) (Carl Roth, Karlsruhe, Germany) to pH 6.8 or with homopiperazine-1,4-bis(2-ethanesulfonic acid) (Sigma-Aldrich, Vienna, Austria) to pH 4.0. The cell density was adjusted photometrically to 5×10^9^ cells ml^−1^. On each single flower, two 10 µl droplets of bacterial suspension were placed, one to the stigmatic surface and one close to the hypanthium resulting in approximately 10^8^ bacterial cells per flower. Mock inoculations were performed with buffer only. Three replicate trees per treatment (pH 4.0 and pH 6.8 with and without *E. amylovora*) were inoculated in the greenhouse at 27/15°C day/night temperature and 80% relative humidity. Three single inoculated flowers per tree were sampled 6, 24, 48 and 72 hours post inoculation (hpi), immediately frozen in liquid nitrogen and kept at −80°C until further processing. Inoculation experiments for flower sampling were performed twice with new trees. For cDNA-synthesis, flowers were transcribed individually in the first (Set 1) and pooled per tree in the second independent experiment (Set 2).

To asses visual symptom development in flowers inoculated at pH 4.0 or pH 6.8, a modified standard test after Pusey, 1997 [41] with detached apple blossoms was applied. In a transparent box 15 detached apple flowers were placed in Eppendorf tubes filled with 1.5 ml 10% sucrose solution and inoculated on the stigmas with a 1 µl drop containing 10^4^ bacterial cells suspended in pH-adjusted water as described above. To attain high humidity 35 ml of 32% glycerine solution was added to each box and closed with a lid. 3 boxes per treatment and 3 boxes with buffer-only inoculated flowers were incubated at approximately 22°C and natural day/night light cycles. After 2 days the flowers were sprayed with pH-adjusted water containing a commercial fungicide. Visual symptom development was analyzed 8 days post inoculation. The detached apple blossom test was performed twice.

### RNA isolation

Total RNA was isolated from sampled apple flowers with petal leafs removed according to the method of Chang et al., 1993 [42]. Isolated RNA was DNAse-treated, checked for quality by gel electrophoresis and A_260_/A_280_ ratio determination, and quantified using a Nanodrop ND-2000 spectrophotometer (Thermo Fisher Scientific, Vienna, Austria). Purified RNA extracts were checked at random for DNA contamination by using RNA extracts as templates in qPCR assays. High quality RNA (5.0 µg per sample in Set 1, 4.2 µg per sample in Set 2) was reverse transcribed with SuperScript VILO cDNA Synthesis Kit according to the manufacturer's protocol (Invitrogen, Carlsbad, California, USA). Thereafter, the obtained cDNA was split for bacterial and plant gene expression analysis.

### Analysis of *E. amylovora* gene expression

Quantitative real-time PCR (qPCR) assays for *E. amylovora* genes *hrpL*, *hrpA*, *hrpN*, *dspA/E*, *amsG*, *recA*, and *gyrA* were established and optimized. Primer sequences were derived from available sequence information in GenBank: *hrpL* (U36244), *hrpA* (U56662), *hrpN* (M92994.3), *dspA/E* (Y13831.1), *amsG* (X77921.1), *recA* and *gyrA* (FN666575.1). To optimize primer concentrations, each primer was tested in 50 nM steps in a concentration range from 50 to 600 nM and annealing temperatures increasing in 1°C steps deviating from the calculated melting temperature by maximally 3°C. Gene-specific PCR products spanning the sequence targeted by the qPCR-primers with 10^4^ copies per reaction were used as templates during optimization. Specific amplification of *E. amylovora* targets was verified by the presence of a specific PCR product in cDNA template from inoculated flowers and absence in mock-inoculated flowers as determined by agarose gel electrophoresis. For analysis, the cDNA transcripts were amplified in a Mastercycler ep realplex (Eppendorf, Hamburg, Germany) with standard reaction conditions: 5 µl cDNA-sample with a final 1∶5 dilution were used in a standard 20 µl-qPCR reaction with Power SYBR Green PCR Master Mix (Applied Biosystems, Darmstadt, Germany) and specific primers (Table S1). Cycling parameters included a 10 min initial denaturation at 95°C followed by 45 cycles consisting of denaturation at 95°C for 15 sec, annealing at primer specific temperature (Table S1) for 25 sec and amplification at 65°C for 25 sec with signal detection. A melting curve analysis was included at the end of each run with a temperature ramp from 60°C to 95°C in 20 min to determine specificity of amplified qPCR products. Each sample was analyzed for bacterial gene expression in triplicate. Quantification of the absolute copy numbers was performed with standard curves of defined dilutions of gene-specific PCR-products in each qPCR run. Data were analyzed with Eppendorf realplex 2.0 software and qPCR efficiencies were between 0.85–1.14 and standard curve R^2^ values above 0.98. No template controls (NTCs) included in each run were always negative. Transcript values were normalized against expression of two standard reference genes: *recA* encoding recombinase A and *gyrA* encoding gyrase subunit A [43].

### Plant gene expression analysis

Expression of the pathogen related protein-1 (*PR-1*; GenBank: AF507974.1) and a gene encoding for the putative proteinase inhibitor Miraculin (*MalMir1*; GenBank: FK938848.1) were determined by qPCR as described in Milčevičová et al. (2010) [44]. For normalization, actin (GenBank: CN915159.1) and GAPDH (GenBank: CN906865.1) were selected as reference genes. Analysis of relative gene expression between inoculated und uninoculated flower samples was done with the software REST 2008 [45].

## Results

The transcriptional timing and coordination of the type III secretion system of *E. amylovora* was investigated for the first time at the site of primary infection, in flowers still attached to the tree. Without wounding, single flowers of the susceptible apple cv. Golden Delicious were manually inoculated with approximately 10^8^ bacterial cells. Bacterial suspension was placed in two droplets at the stigmas and close to the hypanthium (Figure S1). Subsequently, the expression of selected genes essential for type III secretion and necessary for bacterial virulence was monitored. These genes comprised *hrpL* and *hrpA*, and two genes encoding for secreted proteins, *hrpN* and *dspA/E*. Transcript abundances of these genes were measured by newly developed qPCR protocols in reverse transcribed RNA-extracts of whole flowers. To account for pathogen abundance on the flower, expression was normalized against transcript abundance of the reference genes *recA* and *gyrA*.

**Figure 1:**
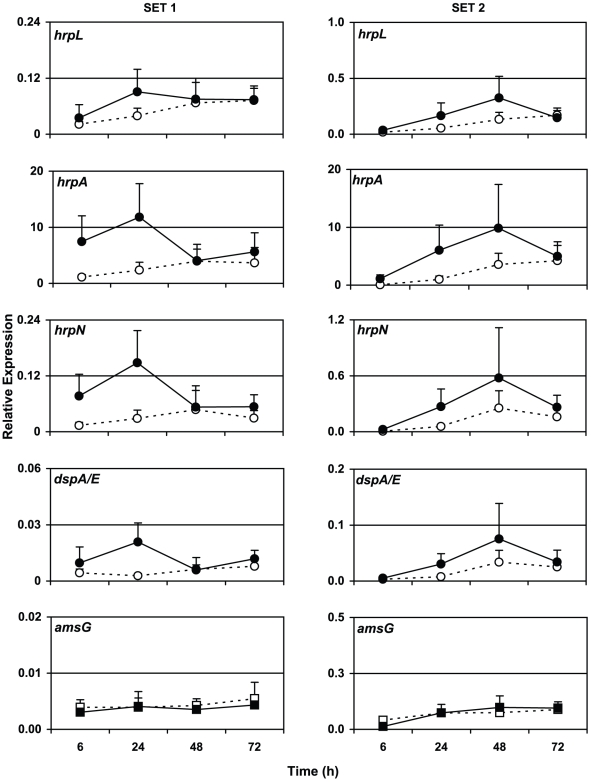
Time-course of *E. amylovora hrp* virulence genes and *amsG* expression upon inoculation on apple flowers. Shown are two independent experiments, Set 1 (5–9 flowers per time point) and Set 2 (3–5 flowers per time point). Normalized expression values of indicated genes represent mean values of flower samples from three replicate trees. Expression is normalized to *recA*. Flowers were inoculated with bacterial suspension buffered to pH 7 (filled symbols) or buffered to pH 4 (open symbols). *hrpL* and *hrpL*–regulated genes are shown as circles whereas *hrp*-independent genes as squares.

No *hrp* expression was observed in non-inoculated flowers (not shown). In two independent inoculation experiments, the mean transcript level of *hrpL*, the main regulator gene of the type III secretion system, increased from low initial expression values 6 hours post inoculation (hpi) to peak expression between 24 to 48 hpi (Figure 1). At 72 hpi, either stable or reduced *hrpL* transcript levels were observed in the first and second experiment, respectively. Parallel to the decline in *hrp* expression, the onset of flower aging was observed at 72 hpi. Petals fell off when touched and stigmas began to discolour (data not shown). The temporal expression pattern observed for *hrpL* was shown concomitantly by the structural gene *hrpA* as well as *hrpN* and *dspA/E* demonstrating a highly coordinated parallel expression during type III secretion (Figure 1). In contrast, expression of *amsG*, the first gene in the operon for amylovoran synthesis, remained for the first 72 h basally low without expression peak as observed for *hrp* genes. Control normalizations for all genes investigated against a second reference gene, *gyr*A, confirmed the observed transcriptional pattern (Figure S2).

**Figure 2:**
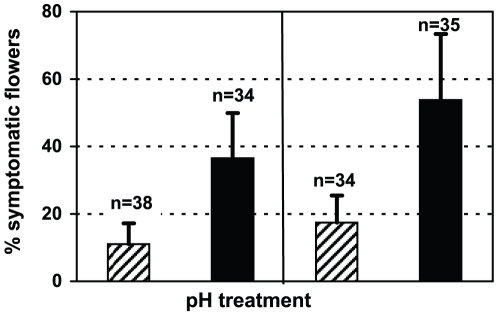
Percentage of flowers with fire blight symptoms after inoculation at acidic or neutral pH conditions. Percentage of symptomatic flowers inoculated with *E. amylovora* suspension adjusted to pH 4 (striped bars) or pH 7 (black bars) in two independent experiments (left and right half of diagram). Bars represent mean percentages of 3 replicate boxes with 11–14 inoculated detached apple flowers. Total flower numbers are indicated above bars. Mean percentages of symptomatic flowers are significantly different between pH treatments on a p<0.05 level (T-test).

To test acidification as one major parameter regulating the expression of the type III secretion system, flower inoculations on additional apple trees were performed in parallel. This time, the bacterial suspension was buffered to pH 4.0 prior to inoculation. As determined by qPCR in these samples, mean transcript levels of *hrpL*, *hrpA*, *hrpN* and *dspA/E* were diminished in comparison to expression levels at neutral pH and slowly increased linearly without peak expression (Figure 1). At the last sampling time point, 72 hpi, mean transcript abundances at pH 4.0 were always lower than values observed at peak expression under neutral conditions. Also at pH 4.0, the expression pattern of *hrpA*, *hrpN* and *dspA/E* followed closely *hrpL* expression with a minor deviation in *hrpN* expression indicating a slight peak expression. In order to test whether molecular expression patterns correlate with visual symptom development, we performed standard infection tests modified after Pusey, 1997 [41] by inoculating detached apple blossoms at the stigmas with 10^4^ *E. amylovora* cells suspended in water buffered to pH 4 or pH 7. The inoculation density in this test system was lower than in greenhouse inoculations (10^8^ cells), because the critical cell density necessary for infection in detached flowers is lower [46]. Evaluation at 8 dpi showed in two independent experiments significantly (T-test, p<0.05) less fire blight symptoms in flowers inoculated and wetted with acidic pH compared to neutral pH (Figure 2).

**Figure 3:**
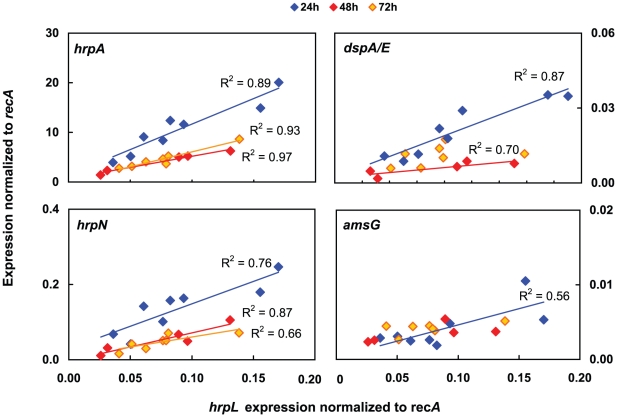
Correlation of gene expression with the transcriptional regulator *hrpL* in single flowers. Expression of *hrpA*, *hrpN*, *dspA/E* and *amsG* is plotted against expression of *hrpL* for the indicated times post inoculation (Set 1). All genes are normalized against *recA* expression. R^2^ values above 0.5 with linear regressions are shown.

To determine the magnitude and relative quantities of *hrp* genes expressed during flower infection, transcript levels were analyzed separately for correlations between genes. After 24 hpi, *hrpA*, *hrpN*, and *dspA/E* transcript abundances were highly correlated with expression of *hrpL* with coefficients of determination being R^2^≥0.7 and R^2^≥0.9 for the first and second experiment, respectively (illustrated for the first experiment in Figure 3). In the early phase of induction (6 hpi) no such correlation with *hrpL* was observed (data not shown). In contrast to *hrp* genes, *amsG* expression showed little correlation with *hrpL* expression with R^2^≤0.6 and R^2^≤0.5 in the first and second experiment, respectively. To find out if *hrpL* expression itself is linearly dependent on the bacterial cell number, normalized *hrpL* expression was compared to the absolute copy number of *recA* transcripts found on the respective flowers. The absence of correlation with *recA* with R^2^≤0.3 (both experiments) suggests that expression of *hrpL* does not directly depend on bacterial abundances at cell densities used for inoculation (illustrated for the first experiment in Figure S3). Comparing absolute transcript abundances during peak expression, *hrpA*∶*hrpN*∶*hrpL*∶*dspA/E* were expressed in a 567∶7∶4∶1 (Set 1) and 131∶8∶4∶1 (Set 2) ratio suggesting that the structural protein pilin is needed in highest abundance. At acidic pH 4.0 the relative proportions of *hrp* transcript abundances were similar to neutral conditions with *hrpA*∶*hrpN*∶*hrpL*∶*dspA/E* in a 849∶10∶14∶1 ratio at 24 hpi (Set 1) and 106∶8∶4∶1 ratio at 48 hpi (Set 2).

**Figure 4:**
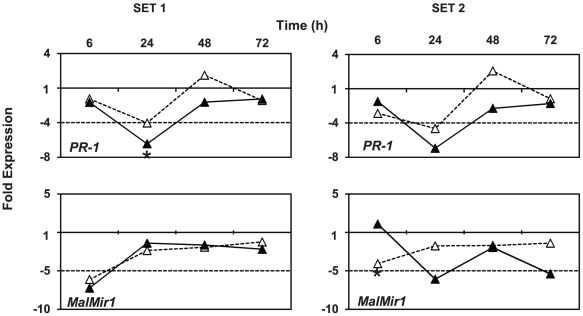
Fold change of *PR-1* and *MalMir1* gene expression in *E. amylovora* inoculated flowers relative to uninoculated flowers. Transcript values for *PR-1* and *MalMir1* are normalized to *actin* and *GAPDH* expression. Flowers were inoculated with bacterial suspension buffered to pH 7 (filled symbols) or buffered to pH 4 (open symbols) and compared to uninoculated (only buffer treated) flowers. The asterisk indicates significant differential expression (p<0.05, REST 2008 analysis).

In parallel, the plant defense response was monitored by expression profiling of the pathogen related protein-1 (*PR-1*) (GenBank: AF507974.1) and the malus miraculin 1 (*MalMir1*) (GenBank: FK938848.1) in the same cDNA samples as used for bacterial expression analysis. Whereas normalized *MalMir1* expression revealed no consistent pattern related to *E. amylovora* inoculation, expression of *PR-1* was transiently downregulated in flowers 24 hpi compared to uninoculated flowers (Figure 4). At 24 hpi, *PR-1* was significantly 6.5-fold less (p<0.05) expressed in the first experiment and 5.5-fold less in the second experiment at neutral pH. At acidic pH, transient downregulation of *PR-1* was less pronounced but in contrast to neutral pH, *PR-1* expression was upregulated 48 hpi (Figure 4).

## Discussion

Type III secretion is essential for *E. amylovora* infection of host vegetative tissue or flowers [17], [47], [48]. However, no expression studies revealed timing of *hrp* gene expression in its entity during the infection process or addressed expression order. Thus, we investigated virulence gene expression for the first time during the major infection mode of *E. amylovora*: floral infection. Because in this case bacteria can invade the healthy flower without the need for artificial wounding, we adopted a non-invasive inoculation method: we placed two droplets of bacterial suspension at flower parts of the susceptible apple ‘Golden Delicious’ where this pathogen can also naturally be detected, the stigmas and close to the hypanthium [4]. This method allows controlling the applied bacterial number per flower and normally yields in our routine greenhouse inoculations 25–48% blighted blossoms in this cultivar.

A characteristic temporal *hrp* expression profile during flower infection included an induction time of approximately 6 hours. This time lies in between time points found for *hrp* inducing medium (3–6 h) and wound-inoculated immature pear (24 h) and comprises time for *hrp* de novo RNA and protein synthesis [10], [29]. For comparison, in the *Pseudomonas syringae*-plant interaction expression of *hrp* genes is induced not until bacteria have reached the intercellular space where direct contact with plant cells is possible [49], [50]. A similar local dependence for full *hrp* expression can be assumed in *E. amylovora* flower infections since bacteria have to migrate first towards nectaries. There, the rising *hrpL* expression would have downregulated flagellar synthesis thereby opposing mobility [51]. The early occurrence of peak expression of *hrp* genes and *dspA/E* in all flowers was intriguing, because it indicates a fast bacterial infection effort rather than sequential attacking. Similarly, a single peak expression of the effector *dspA/E* at 48 hpi was found in *E. amylovora* populations growing epiphytically on apple stigmas [37]. Fast infections might be advantageous, since plant defense is encountered in an initially uninduced state (Figure 4). Notably, even the presence of an avirulent *E. amylovora* strain is detected by the plant and triggers a rapid defense response [52]. Also important in this context is flower age. *E. amylovora* infects successfully flowers 1–3 days old but susceptibility drastically decreases with flower age [2], [53]. This is in good accordance with what we have observed in our greenhouse experiments visually and in gene expression. Petal leaf fall and necrosis of the stigmas were observed from the third day on and concomitantly also expression of the type III secretion system declined 72 h post inoculation. This emphasizes the importance of a fast expression of the type III secretion system especially during early plant invasion by *E. amylovora*. In contrast to the expression profile of the type III secretion system, expression of *amsG*, the first gene in the amylovoran synthesis operon [54], was only basally expressed during early infection. This is in accordance with previous observations that this virulence factor is important later in pathogenicity [55].

When comparing the *E. amylovora hrp*/*dspA/E* gene expression profile with the leaf pathogen *Pseudomonas syringae pv. phaseolicola*, a similar peak expression curve is evident, however, the period of *hrp* expression was shortened to 24 h upon *P. syringae* infiltration into host leaves [56]. Characteristic for both pathogens seems to be that strong *hrp* expression is followed by a decline in expression during the infection. *P. syringae* cells turned their type III secretion off as soon as they had established inside the plant [56] which might be similar for *E. amylovora* but has not been investigated yet. For other plant pathogens with similar type III secretion systems such as *Erwinia herbicola* or *Pantoea stewartii* information about expression profiles during plant invasion are currently not available but could reveal if this expression pattern is common to plant pathogens.

Accumulation of *hrp* transcripts differed between single inoculated flowers indicative for induction to various degrees (Figure 3). These variable transcript levels did not correlate with the bacterial cell number as estimated by abundance of two independent reference gene transcripts (Figure S3), but correlated significantly with *hrpL* transcript abundance. The close co-expression of *hrpA*, *hrpN*, and *dspA/E* with *hrpL* demonstrated by correlation coefficients above 0.7 is consistent with a direct genetic induction of this system through *hrpL* [17]. *hrpL* transcript accumulation itself varied up to 5-fold during peak expression between individual flowers, which might be explained by a different amount of bacteria that reached the hypanthium. We hypothesize that highest expression could be expected in bacterial populations staying at the nectaries or inside the plant whereas lower expression could be expected in epiphytically growing populations. In conclusion, the variable overall expression would explain why even in artificially inoculated flowers, only a portion become infected and shows symptoms later on.

The relative transcript abundances between genes were found in both independent inoculation experiments in the order *hrpA>hrpN>hrpL>dspA/E*. The exceptionally low but efficient amount of *dspA/E* was also previously recognized in transient expression experiments of *dspA/E* in apple leaves, where neither the mRNA nor protein were detectable despite necrosis was elicited [57]. Our results support this conclusion since even with the highly sensitive qPCR method *dspA/E* transcripts were detectable in lowest abundances only. Consistent with the here observed relative transcript abundances, the encoded proteins are secreted in similar proportions into inducing medium [58]. Congruently, expression of the homologous *hrp* genes in *P. syringae* followed closely *hrpL* expression over time and in similar relative quantities with *hrpA*>*hrpZ*>*hrpL*>*avrE* [56]. Together with our data, this suggests that strong upregulation of structurally important transcripts as *hrpN* and *hrpA* are necessary to provide efficient effector placement into flower tissue.

One important question for understanding host susceptibility is how plant defense systems are manipulated by *E. amylovora* during floral infection. We addressed this question by analyzing the expression of two plant genes possibly involved in host defense: a gene encoding for the putative proteinase inhibitor Miraculin (*MalMir1*), which was highly upregulated upon *E. amylovora* shoot infections [59] and the pathogenesis-related protein 1 (*PR-1*), which is a well known indicator for salicylic acid (SA) signaling. The flowers in our experiments were still attached to the living tree to ensure a natural plant defense reaction. For the putative proteinase inhibitor Miraculin encoded by *MalMir1* no consistent expression pattern was observed, which indicates no role in defense against *E. amylovora* in flowers. Contrary, expression of *PR-1* was lowered at 24 hpi in both experiments suggesting a transient suppression caused by *E. amylovora* since no such expression change was observed in mock-inoculated flowers. In *Malus domestica* several *PR*-genes were identified with three different *PR-1*-like genes *PR-1a*, *PR-1b* and *PR-1c* [33]. None of these *PR-1*-like genes were upregulated due to *E. amylovora* inoculation in apple shoots [33] or detached flowers [34]. Our *PR-1* real-time primers specifically target *PR-1a* and we found not only absence of induction but a transient suppression in *PR-1* expression upon *E. amylovora* infection in flowers. Several previous studies presumed manipulation of the SA pathway by this pathogen, however, could not find transcriptional evidence probably due to the temporal limited and transient nature of expression or methodical sensitivity [31], [33]. Also, a recent microarray study did not detect differential expression of *Pr-1* [34], which might have been missed, because the plant response was investigated in flowers which were detached from the plant. However, manipulation of the SA pathway either directly or indirectly, e.g., via the antagonistic jasmonic acid pathway would be a critical function of certain type III effectors for successful host infection [31], [35]. Therefore, we speculate that in our experiments DspA/E caused the observed *PR-1* suppression, since this effector was suggested to modulate basal, probably SA-dependent host plant defense such as callose deposition [31], [32]. We suggest that expression of *dspA/E* had reached already at 24 hpi a threshold level that caused the observed *PR-1* suppression, even though in the second experiment maximal *hrp* expression was only reached at 48 hpi (Figure 1). Further indirect evidence for involvement of DspA/E in SA-defense manipulation is given by delayed *PR1*-expression, when *dspA/E* is transiently expressed in non-host tobacco leaves [57].

Acidification as potential mechanism to prevent new fire blight infections in flowering orchards is discussed since a long time. Several commercially available products are either based solely on this mode of action such as acidic stone meal or are formulated in acidic buffers including desinfectants [60] or acid-tolerant antagonistic yeasts [40]. Recently, natural acidification by the antagonist *Pantoea agglomerans* was suggested as potential additional antagonistic mechanism against *E. amylovora* [61]. The common understanding how acidic pH prevents fire blight infections is mainly derived from the growth-inhibiting effect of pH<5 on *E. amylovora* [40], [62], [63] thereby preventing multiplication on the flower, which is necessary to reach a critical cell density for infection [2]. Also in our experiments *E. amylovora* did not grow below pH 5 (Figure S4). The critical cell density necessary for infection is estimated in fire blight forecasting models to be at least 10^5^–10^6^ colony-forming units (CFU) per flower [64], [65] which is close to the naturally observed *E. amylovora* population densities of 10^6^–10^7^ CFU per flower [2]. Less is known how acidic pH affects the further development of infection when the critical cell density is reached. To investigate *hrp* gene expression at pH 4 and pH 7 without the effect of growth retardation by acidic pH, we applied *E. amylovora* inoculum densities (10^8^ cells/flower) above the threshold density necessary for infection. Interestingly, Wei et al. [10] demonstrated that acidic pH 5.5 is inducing type III secretion in *E. amylovora* in liquid culture, which might hypothetically increase virulence and in consequence would be unwanted in fire blight control. Thus, we addressed the question if a more acidic pH 4 still increases expression of the type III secretion system during flower infection. On a molecular level, expression of *hrp* genes at acidic pH 4 was reduced without a typical peak expression curve as compared to bacterial suspension buffered to pH 7 on flowers (Figure 1). Therefore, in contrast to pH 5.5 the more acidic pH 4 does not induce *hrp* expression anymore. The non-inducing effect of pH 4 is meaningful considering the pH range naturally encountered by plant pathogenic bacteria during infection, which is usually between 5.0–6.5 in the apoplast [66]. However, over time expression of *hrp* genes at acidic pH increased slowly and linearly but never reached peak expression levels observed at neutral pH. On some of the flowers, a minor expression peak for *hrpN* was observed indicating few successful infection events. Thus, under field conditions where flowering time may be prolonged compared to the greenhouse, the pathogen might still be able to infect albeit slower and at lower frequency. Interestingly, in flowers inoculated at acidic pH the plant defensive gene *PR-1* was less suppressed at 24 hpi compared to neutral inoculations (Figure 4). Moreover, a clear upregulation of *PR-1* at 48 hpi indicated that acidification disturbs the bacterial infection progress leading to activation of the plant defense. Regarding visible symptoms on inoculated blossoms, acidification of the bacterial suspension with pH 4 buffered water could reduce (significant at p<0.05; T-test) the number of flowers showing symptoms typically for fire blight (Figure 2). Together with our gene expression data, this indicates that acidification leads to slower and reduced infection rates and might well be an effective measure to reduce fire blight.

In summary, a non-invasive inoculation technique allowed us to study the virulent behaviour of the *E. amylovora* pathogen on the flower and, in parallel, to observe the plant defense reaction in flowers still attached to the living tree. *E. amylovora* expressed key genes for type III secretion, namely the pilin *hrpA*, the putative translocator *hrpN* and the effector *dspA/E* in a narrow time frame of 24–48 hpi and in well-defined ratios under the control of the regulator *hrpL*. No hierarchy for the expression of these genes (this study) or for the encoded secreted proteins was found [58]. This leads to a model where simultaneous expression of the type III components is required for successful infection. The bacterial presence as well as secreted HrpN is recognized by host cells [32], [52], [67], thus concomitant injection of effectors is necessary to counteract elicitation of defense responses. Interestingly, main expression of *hrp* genes coincided with a transient suppression in plant *PR-1* expression at 24 hpi suggesting that *E. amylovora* quickly impacts the major SA-dependent plant defense pathway. This implies that co-transcription of *E. amylovora* structural genes with effectors of the type III secretion system is necessary to outrun plant defense.

## Supporting Information

Figure S1 **Non-invasive inoculation of an apple flower with** ***E. amylovora*** **cell suspension.** One droplet was applied to the stigmatic surface, one close to the hypanthium.
(TIF)

Figure S2 ***E. amylovora hrp*** **virulence genes and** ***amsG*** **expression upon apple flower inoculation.** Shown are expression profiles of indicated genes for two independent experiments, Set 1 (5–9 flowers per time point) and Set 2 (3–5 flowers per time point). Expression values of indicated genes were normalized to *gyrA* expression and represent mean values of flower samples from three replicate trees. Flowers were inoculated with bacterial suspension buffered to pH 7 (filled symbols) or buffered to pH 4 (open symbols). *hrpL* and *hrpL*–regulated genes are shown as circles whereas *hrp*-independent genes as squares.
(EPS)

Figure S3 **Transcript abundances of reference genes as estimate for bacterial abundance compared to relative** ***hrpL*** **expression.** **A** *recA* transcript numbers on single flowers (as estimate for *E. amylovora* cell numbers) plotted against relative expression of *hrpL* (*hrpL*/*recA*) at indicated time points post inoculation. **B** The same transcript values are plotted against *gyrA* transcript numbers and gave virtually identical results.
(EPS)

Figure S4 **Growth curves of** ***E. amylovora*** **295/93 in minimal medium at different pH.** Growth at 26°C was determined as increase in optical density at 630 nm in minimal medium modified after Pusey (1999) [68]: K_2_HPO_4_ 0.7 g/l, KH_2_PO_4_ 0.2 g/l, 10% sucrose, buffered with homopiperazine-1,4-bis(2-ethanesulfonic acid) to pH 4.8, with 2-(N-morpholino)ethanesulfonic acid to pH 5.8, with piperazin-N,N′-bis(2-ethanesulfonic acid) to pH 6.8, and with N-Tris[hydroxymethyl]methyl-3-aminopropanesulfonic acid to pH 7.8 or pH 8.8. Values shown represent the mean of at least 3 independent trials with 5 replicates and standard deviations (not visible for pH 4.8).
(EPS)

Table S1 **Primer sequences and PCR conditions used in qPCR analyses and standard PCR.**
(DOC)
