# Supplementary figures and images for: Erwinia amylovora Expresses Fast and Simultaneously hrp/dsp Virulence Genes during Flower Infection on Apple Trees
Source: PLoS One. 2012 Mar 6;7(3):e32583. doi: 10.1371/journal.pone.0032583 (PMC3295760; doi:10.1371/journal.pone.0032583)

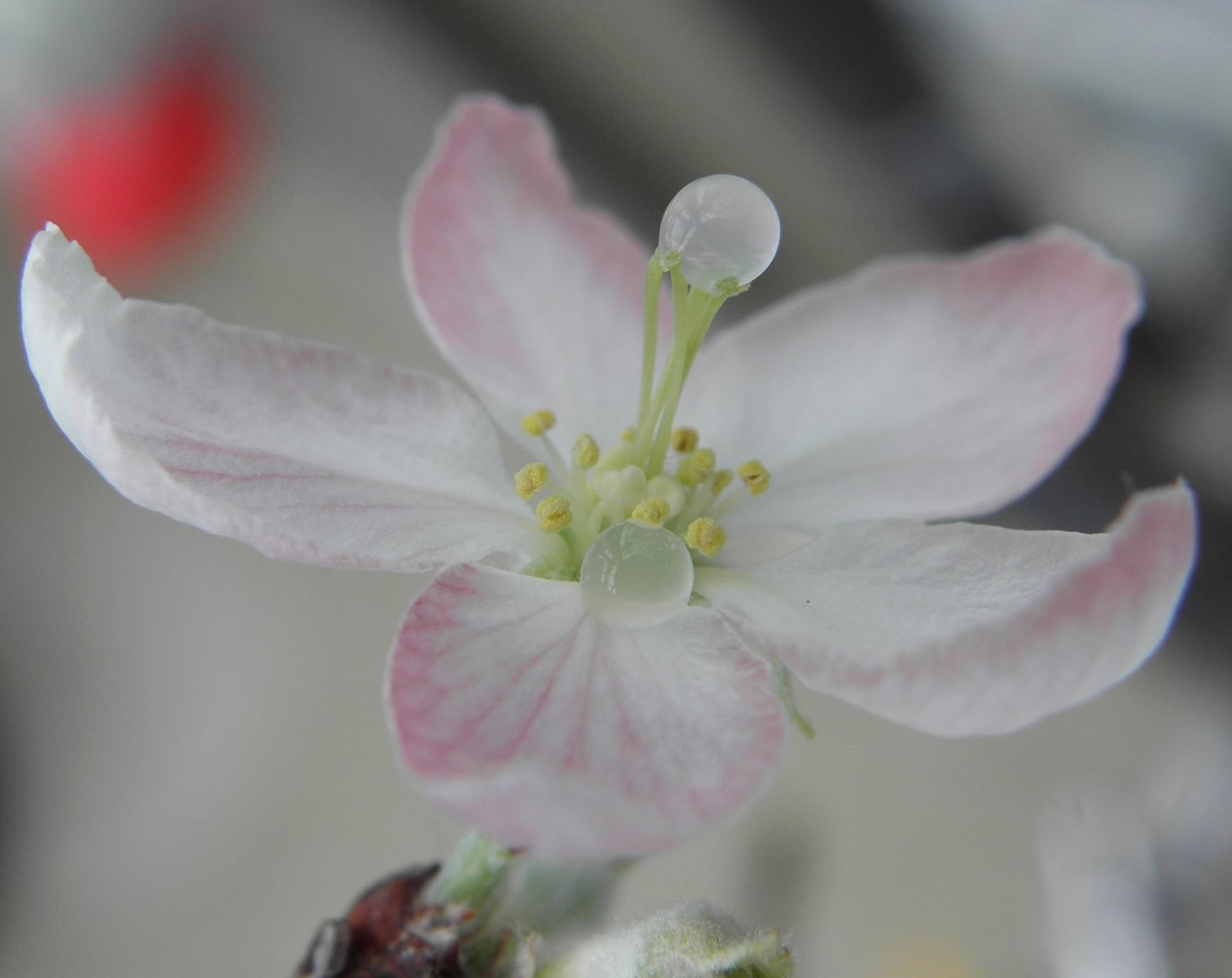

Supplement: Figure S1 — Non-invasive inoculation of an apple flower with E. amylovora cell suspension. One droplet was applied to the stigmatic surface, one close to the hypanthium. (TIF) [file pone.0032583.s001.tif]
