# Supplementary material for: Erwinia amylovora Expresses Fast and Simultaneously hrp/dsp Virulence Genes during Flower Infection on Apple Trees
Source: PLoS One. 2012 Mar 6;7(3):e32583. doi: 10.1371/journal.pone.0032583 (PMC3295760; doi:10.1371/journal.pone.0032583)
Supplement: Table S1 — Primer sequences and PCR conditions used in qPCR analyses and standard PCR. (DOC) [file pone.0032583.s005.doc]

Table S1. Primer sequences and PCR conditions used in qPCR analyses and standard PCR.

| name | primer sequence 5'–3' | concentration [nM] | annealing temperature °C |
| --- | --- | --- | --- |
| ***E. amylovora* primers for qPCR targets** | |  |  |
| hrpL fw | GACGATCCCAGCCATATCAC | 400 | 59 |
| hrpL rev | ACCAGCATGTTCAACAGACG | 400 | 59 |
| hrpA fw | TACAAGCGCAAGCACTTCAG | 400 | 60 |
| hrpA rev | GAGTCCATTTTGCCATCCAG | 400 | 60 |
| hrpN fw | AATGATATCGGTACGCACAGC | 400 | 60 |
| hrpN rev | CCATGAACTGACCGATTTCC | 400 | 60 |
| dspA/E fw | GCGCCACAGCCAGATTTGC | 600 | 60 |
| dspA/E rev | AGCACTCCCTGATGTTGACC | 300 | 60 |
| amsG fw | GCTTTATGGCACGGATATGG | 300 | 62 |
| amsG rev | CCAACGAGATCGAAGGTACG | 300 | 62 |
| recA fw | AAATTATCGATGTCCACGCC | 600 | 60 |
| recA rev | AAACCACCCTGACTTTGCAG | 600 | 60 |
| gyrA fw | TCCAGCGGCAGCAGGTTGAC | 300 | 63.3 |
| gyrA rev | AAGGTAAGTCGGCGGCACGG | 300 | 63.3 |
| ***E. amylovora* primers used for qPCR standard generation** | | | |
| hrpL fw o | GGAAGACGATATCATCCTGACTC | 10000 | 58 |
| hrpL rev o | TGCAATAGCCTGATAACTGCC | 10000 | 58 |
| hrpA fw o | GGTATGGCAGGCAGTTCAC | 10000 | 58 |
| hrpA rev o | GCCAGAGTTTATGGACTTAGAAGC | 10000 | 58 |
| hrpN fw o | ccggtggactaccagcag | 10000 | 60 |
| hrpN rev o | tttgcccatgatttgtcatc | 10000 | 60 |
| dspA/E fw o | Gaagccgttgtacgagatgc | 10000 | 59 |
| dspA/E rev o | cgattgacgttaaagctctgg | 10000 | 59 |
| amsG fw o | tatcgtggcggttgaatatg | 10000 | 59 |
| amsG rev o | tcgagcgcaacttaagacac | 10000 | 59 |
| recA fw o | CGGTATCTGGCTGAGAGCAC | 10000 | 58 |
| recA rev o | GCATCGTTGAAATTTACGGC | 10000 | 58 |
| gyrA fw o | CGATCAGTTCGGTGACAAAC | 10000 | 59 |
| gyrA rev o | TGCAGTAATACGCTCGTTGG | 10000 | 59 |
| ***Malus domestica* primers (host plant)** | |  |  |
| Pr-1 fw | CTTGACGTGGGATGACAATG | Milcevicova et al. 2010 [44] | |
| Pr-1 rev | AGTGCTCATGGCAAGGTTTT | Milcevicova et al. 2010 [44] | |
| Malmir1 fw | CAGTCTCACCCACCTTCCAT | Milcevicova R., 2009 [59] | |
| Malmir1 rev | TTTTGTTGAGGGGGAAACTG | Milcevicova R., 2009 [59] | |
